# Comparison of intravascular ultrasound guided versus angiography guided drug eluting stent implantation: a systematic review and meta-analysis

**DOI:** 10.1186/s12872-015-0144-8

**Published:** 2015-11-17

**Authors:** Yao-Jun Zhang, Si Pang, Xiao-Yun Chen, Christos V. Bourantas, Dao-Rong Pan, Sheng-Jie Dong, Wen Wu, Xiao-Min Ren, Hao Zhu, Shun-Yi Shi, Javaid Iqbal, Bill D. Gogas, Bo Xu, Shao-Liang Chen

**Affiliations:** Department of Cardiology, Nanjing First Hospital, Nanjing Medical University, Nanjing, China; Medical School of Southeast University, Nanjing, China; University College of London Hospital NHS Foundation Trust, University College of London, London, UK; Department of the Joint and Bone Surgery, Yantaishan hospital, Yantai, Shandong province China; University of Sheffield, Sheffield, UK; Andreas Gruentzig Cardiovascular Center, Emory University School of Medicine, Atlanta, GA USA; Department of Cardiology, Fu Wai Hospital, National Center for Cardiovascular Diseases, Chinese Academy of Medical Science, Beijing, China; Nanjing First Hospital, Nanjing Medical University, No. 68, Changle Road, 210006 Nanjing, Jiangsu China

**Keywords:** Intravascular ultrasound, Angiography, Drug-eluting stent, Meta-analysis

## Abstract

**Background:**

Intravascular ultrasound (IVUS) can be a useful tool during drug-eluting stents (DES) implantation as it allows accurate assessment of lesion severity and optimal treatment planning. However, numerous reports have shown that IVUS guided percutaneous coronary intervention is not associated with improved clinical outcomes, especially in non-complex patients and lesions.

**Methods:**

We searched the literature in Medline, the Cochrane Library, and other internet sources to identify studies that compare clinical outcomes between IVUS-guided and angiography-guided DES implantation. Random-effects model was used to assess treatment effect.

**Results:**

Twenty eligible studies with a total of 29,068 patients were included in this meta-analysis. The use of IVUS was associated with significant reductions in major adverse cardiovascular events (MACE, odds ratios [OR] 0.77, 95 % confidence intervals [CI] 0.71-0.83, *P* < 0.001), death (OR 0.62, 95 % CI 0.54-0.71, *p* < 0.001), and stent thrombosis (OR 0.59, 95 % CI: 0.47-0.73, *P* < 0.001). The benefit was also seen in the repeated analysis of matched and randomized studies. In stratified analysis, IVUS guidance appeared to be beneficial not only in patients with complex lesions or acute coronary syndromes (ACS) but also patients with mixed lesions or presentations (MACE: OR 0.69, 95 % CI: 0.60-0.79, *p* < 0.001, OR 0.81, 95 % CI 0.74-0.90, *p* < 0.001, respectively). By employing meta-regression analysis, the benefit of IVUS is significantly pronounced in patients with complex lesions or ACS with respect to death (*p* = 0.048).

**Conclusions:**

IVUS guidance was associated with improved clinical outcomes, especially in patients with complex lesions admitted with ACS. Large, randomized clinical trials are warranted to identify populations and lesion characteristics where IVUS guidance would be associated with better outcomes.

## Background

Although there is evidence about the efficacy of drug-eluting stents (DES) for treating coronary artery disease, patients are not free of events as there is a considerable risk of restenosis and stent thrombosis (ST) after DES implantation [1]. Intravascular ultrasound (IVUS) with its high resolution appears as a useful tool for evaluating lesion severity, optimizing stent implantation and subsequently reducing adverse cardiovascular events [2, 3]. However, due to lack of universally identical IVUS guidance criteria and large randomized clinical trials, the use of IVUS for guiding DES implantation has been a controversial issue among the interventionlists, with many of them believing that its use increases cost and has only a limited clinical benefit.

The results observed in a prespecified substudy of ADAPT-DES (Assessment of Dual Antiplatelet Therapy With Drug-Eluting Stents) showed that IVUS guidance was strongly associated with lower incidences of ST, myocardial infarction (MI) and major adverse cardiac events (MACE) in all-comers population at 1 year follow-up [4]. The improved outcomes noted in the IVUS-guided group have been attributed to the longer and lager stents used in the IVUS guidance group. However, a recent large observational study reported that IVUS-guided percutaneous coronary intervention (PCI) was not associated with improved long-term survival compared with standard angiography-guided PCI [5]. The differences in outcomes noted in different studies reflect the undefined role of IVUS during PCI in clinical practice. Although meta-analyses have shown better outcomes in patients undergoing IVUS guided PCI [6–8], to date, there are limited data comparing IVUS guidance with angiography guidance PCI in patients with complex lesions or acute coronary syndromes (ACS).

Therefore, in this study we update our previous meta-analysis and perform subgroup analysis with matched and randomized studies and assess the effect on clinical outcomes of IVUS guidance. We further investigate whether IVUS guided DES implantation is associated with a greater benefit in patients with complex lesions or ACS.

## Methods

### Data sources and search strategy

We conducted the meta-analysis in accordance with the PRISMA (Preferred Reporting Items for Systematic reviews and Meta-Analyses) statement for studies that evaluate healthcare interventions [9]. We searched the literature in Medline, the Cochrane Library from January 1995 to October 2014, using combinations of the medical subject headings “ultrasound, intravascular”, “IVUS”, “IVUS-guided”, “angiography”, “angiography-guided”, “drug-eluting stent” and “DES”. We used the Science Citation Index as a cross reference to include studies that met the search criteria. We also searched potential studies from the conference proceedings of the American College of Cardiology, the American Heart Association, the European Society of Cardiology and the Transcatheter Cardiovascular Therapeutics. Additionally, we reviewed the reference of the selected articles and earlier meta-analyses for related documents.

### Study identification and data extraction

Two investigators (PS and CXY) independently conducted the literature search, data extraction and quality evaluation through a standard method. Differences were resolved by consensus with third investigator (ZYJ). Studies were included in the current meta-analysis if they met the following predetermined criteria [4, 10–28]: (1) clinical research published in peer-reviewed journals with complete data; (2) comparison of IVUS- versus angiography-guided DES implantation; and (3) at least 6 months follow up. Studies that included bare metal stents (BMS) and DES implantation and did not provide separately the DES data were excluded. Two investigators (PS and CXY) extracted the baseline information, which included the study name, study design, sample size, follow-up duration, patients’ baseline characteristics (mean age, sex distribution, and risk factors), lesion and procedural characteristics, and clinical outcomes. The Newcastle-Ottawa-Scale (NOS) scale was used for quality assessment, including assessment of selection of the exposed and unexposed cohort, comparability of the two cohorts, and outcome assessment [29]. The qualities of randomized trials were assessed by the Jadad score [30].

### Clinical endpoints

The endpoints of the present analysis included: (1) all-cause death (in 2 studies [11, 13] that only reported cardiac death was included instead), (2) MACE, (3) ST (definite or probable ST, according to the definition of the Academic Research Consortium), (4) MI (in 1 study [14] only the Q-wave MI was reported while others reported both non-Q-wave MI and Q-wave MI), (5) target vessel revascularization (TVR), and (6) target lesion revascularization (TLR). Repeated-analyses were performed in patients with complex lesions or ACS compared to mixed lesions or any presentations and among propensity-matched and randomized studies.

### Statistical analysis

The guideline of the Cochrane Handbook for Systematic Reviews of Interventions was implemented in this meta-analysis [31]. Standard data extraction and calculation were used to improve efficiency and reliability of the analysis [32]. Random-effects model was adopted to measure overall treatment effect expressed as odds ratios (OR) and 95 % confidence intervals (CI). Forest plots were generated for graphical presentations of clinical outcomes with IVUS- versus angiography-guided groups. We assessed heterogeneity of the study using chi-square tests (*p* > 0.1 showed no significant heterogeneity among studies) and *I*^*2*^ statistic (*I*^*2*^ > 25 %, >50 %, >75 % showed low, moderate and severe heterogeneity, respectively). All p-values were two-tailed and the statistical significance was considered at <0.05. In case there was heterogeneity among the studies, we conducted sensitivity analyses to clear the source of the heterogeneity. We tested the interaction between patients with complex lesions or ACS versus patients with mixed lesions or any clinical presentations by means of weighted least squares random-effect meta-regression, with weighting provided by the inverse of the variance of each study, patients with complex lesions or ACS (coded as 1 versus patients with mixed or any clinical presentations coded as 0) as random factor, and the natural logarithm of the individual OR as dependent variable [29]. The Egger’s linear regression test was performed for asymmetry of the publication or reporting bias [33]. All statistical analysis was performed with STATA 12.0 (Stata Corp, College Station, TX, USA).

### Ethics

This meta-analysis didn’t require ethical approval.

## Results

### Inclusion of studies

**Fig. 1:**
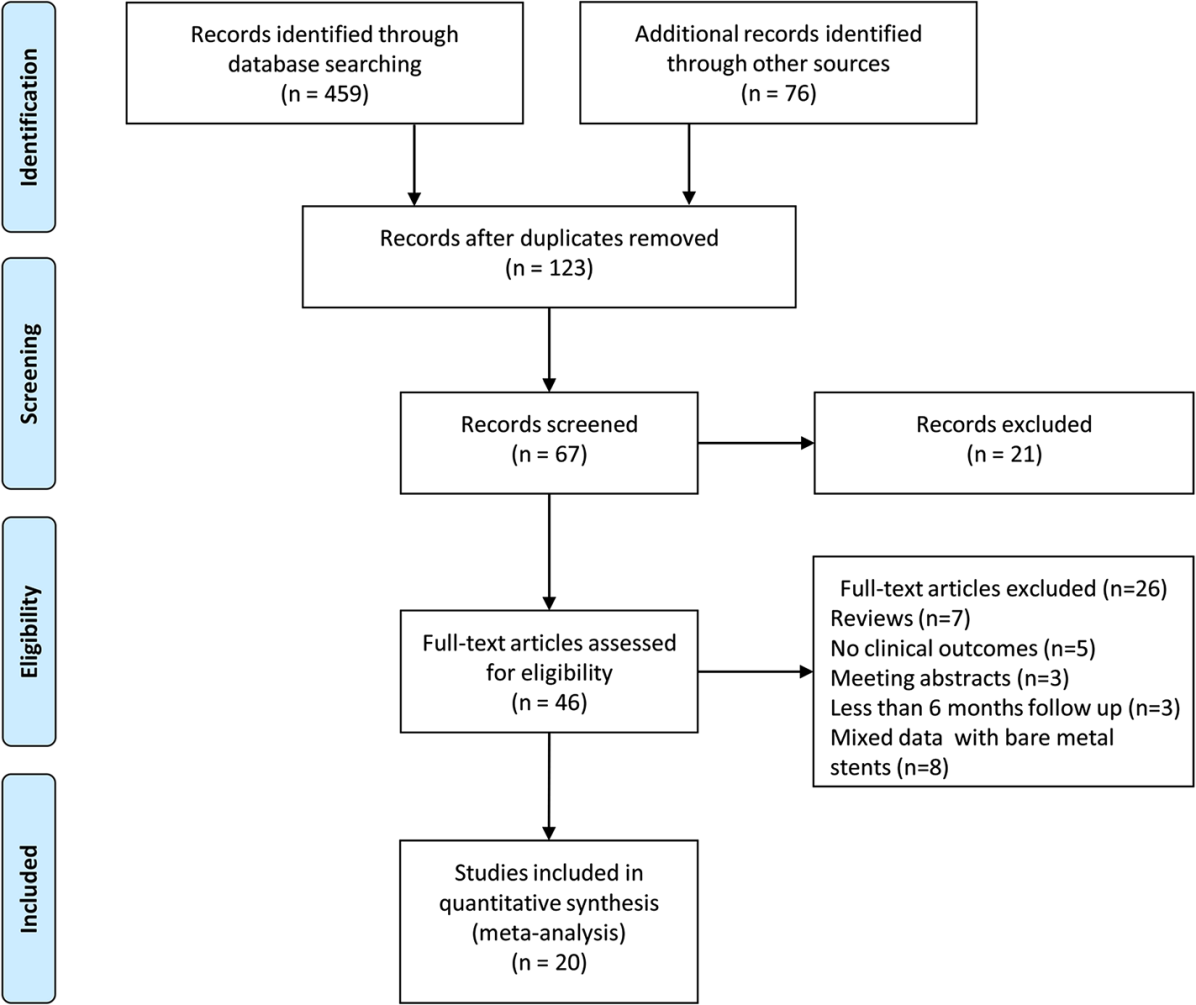
Flow diagram of meta-analysis

In total, twenty eligible studies were included in this meta-analysis (Fig. 1). Out of 20 studies, 3 studies were prospective, randomized trials [10–12], and 17 were observational registries [4, 13–28]. In addition, 9 of the included studies had performed sub-analysis after propensity score matching [14, 15, 17, 18, 20, 21, 26–28]. Therefore, 13 studies enrolled only patients with complex lesions or ACS, including 4 for left main disease [13, 15, 26, 28], 3 for bifurcation [16, 17, 21], 1 for chronic total occlusion (CTO) [27], 1 for long lesion [12], 1 for ST-segment elevation MI [24], and 3 for combined complex lesions [10, 11, 24].

Out of 29,068 patients included in this study, 13,552 (46.6 %) patients underwent IVUS-guided DES implantation and 15,516 (53.4 %) angiography-guided DES implantation. The study characteristics are summarized in Table 1. The mean weighted follow-up was 20.8 months. Lesion and procedural characteristics are shown in Table 2.

**Table 1:** Study design and baseline characteristics

Study	Year	Design	Sample size	F/U Months	Age years	Male	DM	Hyperlipidemia	LVEF, %	Renal insufficiency	Smoker	Study quality (Max = 9)
P Agostoni [13]	2005	Observational	24/34	14	62/64	15/25	9/10	15/23	52/44	NA/NA	4/7	7
P Roy [14]	2008	Observational	884/884	12	66/66	613/619	317/304	762/770	47/48	110/112	186/181	9
SJ Park [15]	2009	Observational	145/145	36	64/65	102/102	49/49	42/44	60/61	7/6	28/30	9
SH Kim [16]	2010	Observational	308/112	48	59/60	221/80	61/24	134/99	60/59	3/1	109/40	8
J Jakabcin [10]	2010	RCT	105/105	18	59/60	77/75	44/47	66/69	NA/NA	NA/NA	42/37	4^a^
JS Kim [17]	2011	Observational	487/487	36	62/62	324/326	155/162	168/170	60/59	15/15	106/111	9
BE Claessen [18]	2011	Observational	631/873	24	64/65	469/652	190/316	533/740	NA/NA	54/97	70/94	9
SH Hur [19]	2011	Observational	2765/1816	36	59/62	1982/1240	622/463	1305/1108	59/57	83/105	979/636	7
KW Park [20]	2012	Observational	619/802	12	62/63	393/524	233/309	468/610	NA/NA	NA/NA	147/233	8
SL Chen [21]	2012	Observational	324/304	12	63/65	261/227	60/54	108/304	61/60	NA/NA	147/154	8
ADAPT-DES [4]	2013	Observational	3349/5234	12	63/64	2457/3901	1048/1735	2287/4093	NA/NA	536/894	851/1088	9
Chieffo A [11]	2013	RCT	142/142	24	64/64	117/109	34/38	100/109	55/56	NA/NA	49/44	4^a^
RESET [12]	2013	RCT	269/274	12	63/64	177/150	85/82	165/165	55/54	NA/NA	58/47	5^a^
YJ Youn [22]	2011	Observational	125/216	36	60/61	93/136	34/71	28/24	45/48	3/6	94/125	8
YW Yoon [23]	2013	Observational	662/912	12	61/63	428/592	184/270	403/512	NA/NA	NA/NA	167/236	8
SG Ahn [24]	2013	Observational	49/36	24	65/65	30/22	13/11	14/9	54/56	5/2	16/14	7
IRIS-DES [25]	2013	Observational	1616/1628	24	62/64	1115/1034	500/516	645/548	60/59	48/57	492/478	7
Hernandez [26]	2014	Observational	505/505	36	66/67	404/397	183/175	314/284	55/55	35/31	148/161	8
SJ Hong [27]	2014	Observational	206/328	24	62/63	159/234	62/124	89/116	NA/NA	NA/NA	58/93	9
XF Gao [28]	2014	Observational	337/679	12	66/67	274/526	109/232	228/487	59/57	88/214	111/230	9

Data are presented as IVUS guidance/ angiography guidance. The Newcastle-Ottawa-Scale was used for quality assessment of observational studies

*Abbreviation*: *DM* diabetes mellitus; *F/U* follow-up; *LVEF* left ventricular ejection fraction; *MI* myocardial infarction; *NA* not available; *RCT* randomized controlled trials

^a^The qualities of included randomized trials were assessed by the Jadad score

**Table 2:** Patient, lesion, and procedural characteristics

Study	Lesion number	LM	LAD	LCX	RCA	Ostial lesion	Stent number	Stent diameter	Stent length
P Agostoni [13]	NA/NA	24/34	0/0	0/0	0/0	7/3	1.5/1.4	3.2/3.2	27/23
P Roy [14]	1.7/1.7	26/30	427/433	320/305	446/450	50/48	1.5/1.5	3.05/3.09	20.7/20.1
SJ Park [15]	NA/NA	145/145	0/0	0/0	75/80	61/62	1.23/1.24	NA/NA	35.2/35.6
SH Kim [16]	1.4/1.2	NA/NA	NA/NA	NA/NA	NA/NA	61/9	NA/NA	NA/NA	34/26
J Jakabcin [10]	1.2/1.2	3/4	59/57	12/16	30/25	NA/NA	NA/NA	NA/NA	23.6/22.1
JS Kim [17]	NA/NA	17/19	404/402	63/63	20/22	NA/NA	1.3/1.2	NA/NA	NA/NA
BE Claessen [18]	1.9/1.8	30/20	349/321	226/307	165/316	55/59	NA/NA	3.1/3.0	23.5/24.5
SH Hur [19]	NA/NA	232/45	1628/904	340/390	686/612	312/84	1.7/1.6	3.3/3.1	38.6/36.7
KW Park [20]	1.4/1.3	0/0	455/502	171/250	227/315	NA/NA	1.3/1.2	3.19/3.06	30.7/23.0
SL Chen [21]	NA/NA	137/83	129/186	44/26	14/9	NA/NA	1.26/1.20	3.25/3.16	32.7/30.5
ADAPT-DES [4]	1.48/1.52	146/171	NA/NA	NA/NA	NA/NA	NA/NA	1.73/1.71	NA/NA	33.6/31.7
Chieffo A [11]	NA/NA	NA/NA	NA/NA	NA/NA	NA/NA	NA/NA	NA/NA	2.95/2.86	23.9/23.2
RESET [12]	NA/NA	0/0	167/185	41/35	61/54	NA/NA	NA/NA	NA/NA	32.4/32.3
YJ Youn [22]	NA/NA	1/2	75/101	7/21	42/92	NA/NA	1.4/1.2	3.18/3.03	34.8/29.5
YW Yoon [23]	NA/NA	0/0	441/566	131/255	163/344	NA/NA	NA/NA	NA/NA	20.4/20.1
SG Ahn [24]	NA/NA	0/0	29/16	6/2	14/18	8/2	2.8/2.2	3/2.87	74/66
IRIS-DES [25]	NA/NA	148/26	1019/958	NA/NA	NA/NA	NA/NA	1.44/1.16	3.28/3.1	35.5/26.9
Hernandez [26]	1.47/1.5	505/505	NA/NA	NA/NA	NA/NA	151/145	NA/NA	NA/NA	NA/NA
SJ Hong [27]	NA/NA	6/4	91/123	34/75	NA/NA	NA/NA	1.7/1.42	2.96/2.83	44.6/36.9
XF Gao [28]	1.2/1.3	337/679	224/479	125/324	146/369	32/59	1.5/1.4	3.5/3.4	35.4/33.3

Data are presented as IVUS guidance/ angiography guidance

*Abbreviation*: *LM* left main coronary artery; *LAD* left anterior descending artery; *LCX* left circumflex artery; *RCA* right coronary artery; *NA* not available

### Effect of IVUS guidance on clinical outcomes

**Fig. 2:**
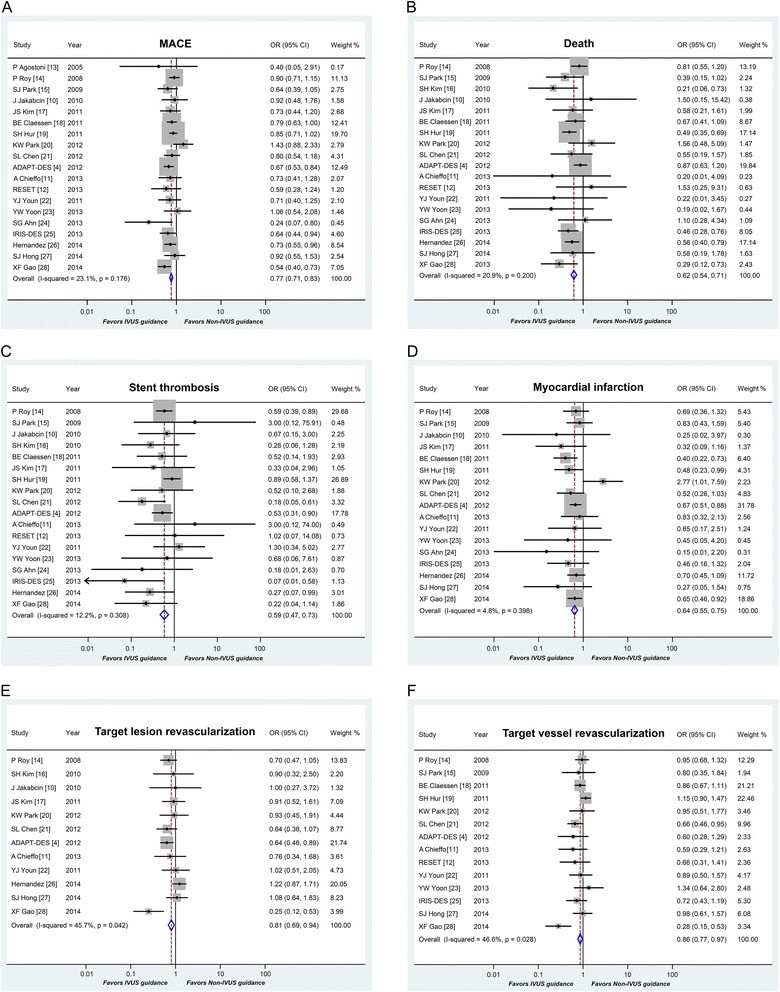
Clinical outcomes after DES implantation with IVUS versus angiography guidance. Abbreviation: MACE = major adverse cardiac events; OR = odds ratios; CI = confidence intervals

MACE were reported in 19 of the 20 included studies. The summary result was in favor of IVUS-guided DES implantation in risk of MACE (OR 0.77, 95 % CI 0.71-0.83, *P* < 0.001; Fig. 2a). Statistical analysis did not show significant heterogeneity (heterogeneity chi-square = 23.40, I^2^ = 23.1 %, *p* = 0.176).

IVUS guidance was associated with a significantly low risk of mortality, compared with angiography guidance (OR 0.62, 95 % CI 0.54-0.71, *p* < 0.001; Fig. 2b). Evidence of statistical heterogeneity was not observed among the studies (heterogeneity chi-square = 22.77, I^2^ = 20.9 %, *p* = 0.200).

The occurrence of definite/probable ST was reported in 19 studies. IVUS guidance appeared to be associated with a significantly low incidence of ST (OR 0.59, 95 % CI: 0.47-0.73, *P* < 0.001; Fig. 2c). There is no statistical heterogeneity in these studies (heterogeneity chi-square = 19.37, I^2^ = 12.2 %, *p* = 0.308).

MI was reported in 18 studies and the pooled result showed that IVUS guidance was associated with a significantly low risk of MI (OR 0.64, 95 % CI: 0.55-0.75, *P* < 0.001; Fig. 2d). The pooled OR for TLR associated with IVUS guidance versus angiography guidance was 0.81 (95 % CI: 0.69-0.94, *P* = 0.005; Fig. 2e) while the pooled OR for TVR was 0.86 (95 % CI: 0.77-0.97, *P* = 0.012; Fig. 2f). Statistical heterogeneity was not found in MI (heterogeneity chi-square = 16.80, I^2^ = 4.8 %, *p* = 0.398), but there was heterogeneity in TLR and TVR (heterogeneity chi-square = 20.27, I^2^ = 45.7 %, *p* = 0.042; heterogeneity chi-square = 24.33, I^2^ = 46.6 %, *p* = 0.028, respectively).

### Outcomes in propensity-matched and randomized studies

**Fig. 3:**
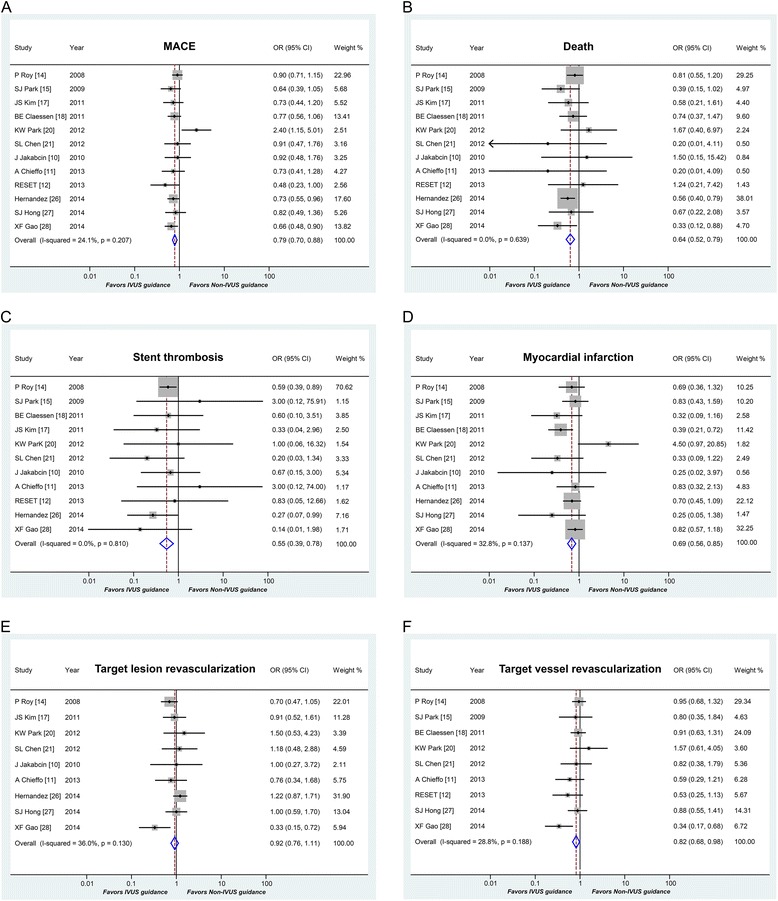
Outcomes in propensity-matched and randomized studies. Abbreviation: MACE = major adverse cardiac events; OR = odds ratios; CI = confidence intervals

In the repeated analysis of propensity-matched and randomized studies, a total of 8,331 patients were included. Repeated analysis confirmed that IVUS guidance was associated with a significant reductions in MACE (OR 0.79, 95 % CI: 0.70-0.88, *P* < 0.001, Fig. 3a), death (OR 0.64, 95 % CI: 0.52-0.79, *P* < 0.001, Fig. 3b), ST (OR 0.55, 95 % CI: 0.39-0.78, *P* = 0.001, Fig. 3c), MI (OR 0.69, 95 % CI: 0.56-0.85, *P* < 0.001, Fig. 3d), and TVR (OR 0.82, 95 % CI: 0.68-0.98, *P* = 0.028, Fig. 3f). No statistical difference was observed in TLR (OR 0.92, 95 % CI: 0.76-1.11, *P* = 0.377, Fig. 3e).

### Stratified analysis in patients with complex lesions or ACS

**Fig. 4:**
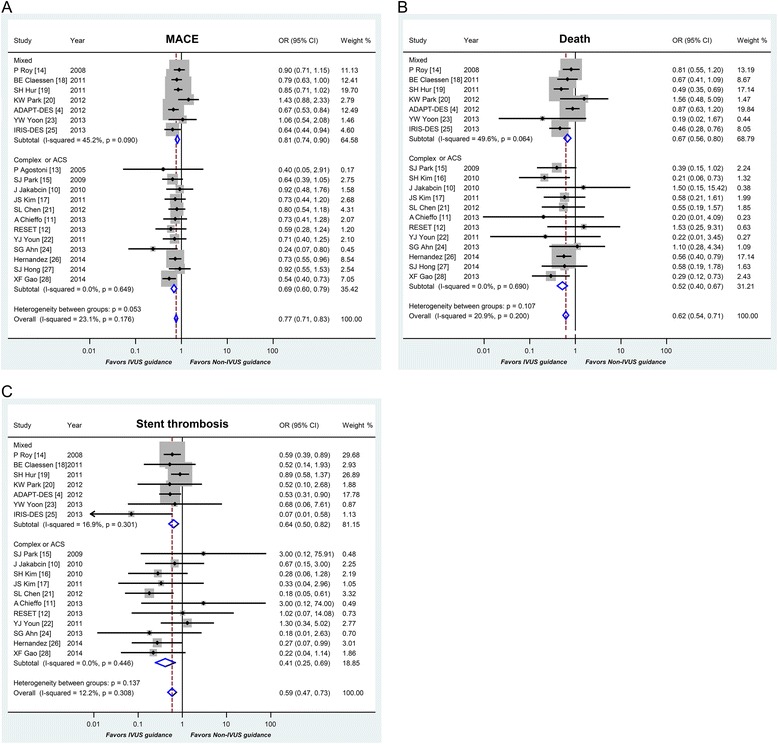
Stratified analysis in patients with complex lesions or acute coronary syndromes. Abbreviation: MACE = major adverse cardiac events; OR = odds ratios; CI = confidence intervals

Sub-analysis was performed to compare IVUS-guided versus angiography-guided PCI with DES for patients with complex lesions or ACS. Thirteen studies with 6,393 patients were eligible for this sub-analysis. IVUS guidance was associated with a low risk of MACE (OR 0.69, 95 % CI: 0.60-0.79, *p* < 0.001), death (OR 0.52, 95 % CI: 0.40-0.67, *p* < 0.001), and ST (OR 0.41, 95 % CI: 0.25-0.69, *p* = 0.001) for patients with complex lesions or ACS, when compared to patients with mixed lesions or any clinical presentation (MACE [OR 0.81, 95 % CI: 0.74-0.90, *p* < 0.001], death [OR 0.67, 95 % CI: 0.56-0.80, *p* < 0.001], and ST [OR 0.64, 95 % CI: 0.50-0.82, *p* < 0.001], respectively) (Fig. 4). By employing meta-regression analysis, the benefit of IVUS guidance is significantly pronounced in patients with complex lesions or ACS with respect to death (*p* = 0.048).

### Sensitivity analyses and publication bias

**Fig. 5:**
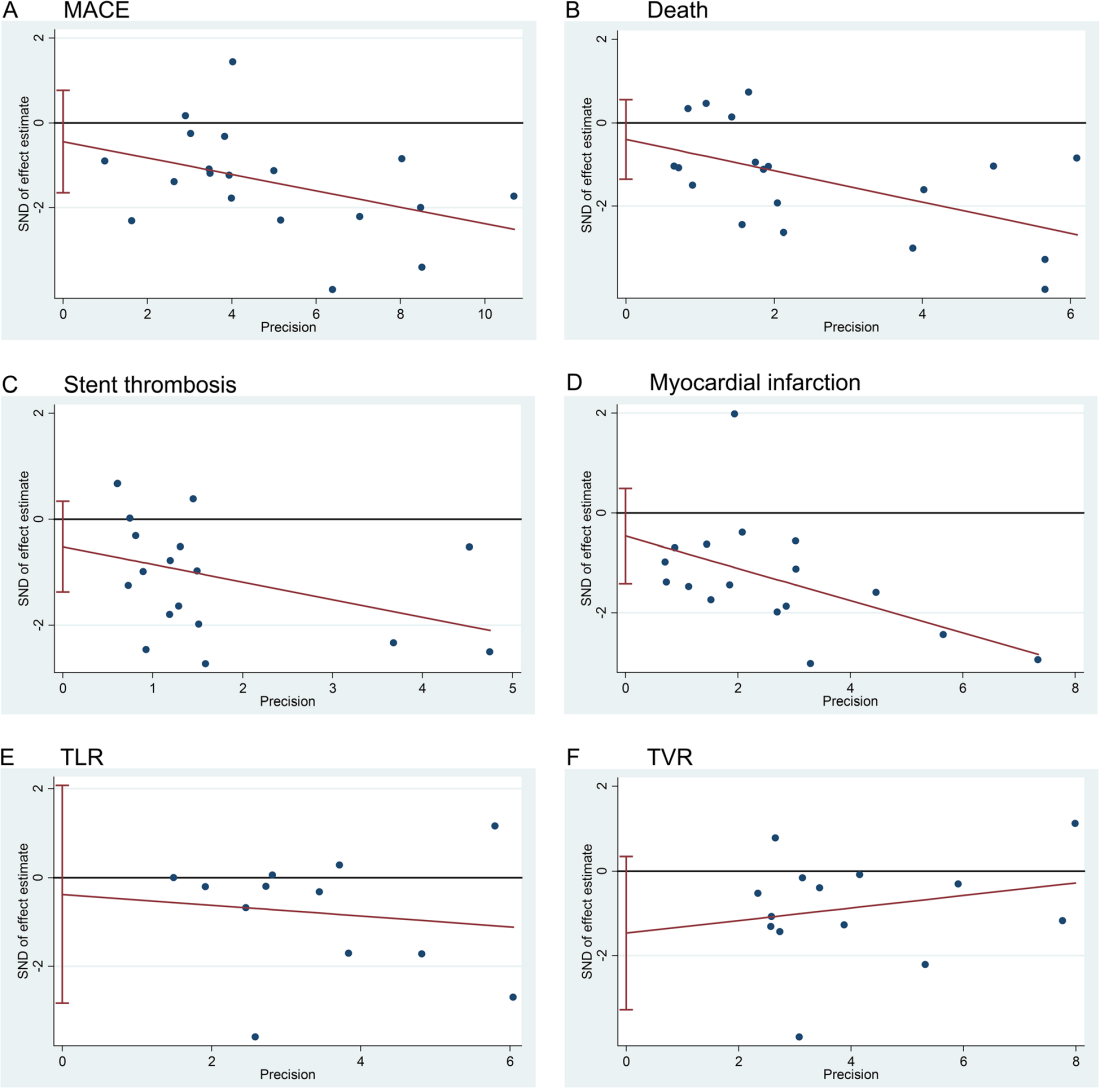
Publication bias. Abbreviation: MACE = major adverse cardiac events; TLR = target lesion revascularization; TVR = target vessel revascularization; OR = odds ratios; CI = confidence intervals

Sensitivity analyses of the lesion subsets did not change the reported results. There was no evidence of publication bias through the Egger’s linear regression analysis (*p* = 0.455 for MACE, *p* = 0.395 for death, *P* = 0.217 for ST, *p* = 0.319 for MI, *p* = 0.738 for TLR, *P* = 0.103 for TVR, Fig. 5). Assessment of publication bias using the logarithm of relative risk showed a symmetric funnel plot and confirmed no evidence of publication bias.

## Discussion

This meta-analysis involving 29,068 patients has shown that IVUS guidance for DES implantation was associated with significantly improved clinical outcomes, when compared with angiography guidance. Similar results were observed in the repeated-analyses of matched and randomized studies. IVUS guidance appeared to have a more beneficial effect in patients with complex lesions or ACS than patients with mixed lesions or presentations with respect to death.

The value of IVUS in guiding DES implantation is still controversial. IVUS-guided PCI could result in larger minimum luminal diameter (MLD) and reduce the incidence of strut malapposition, but does not appear to improve clinical outcomes compared to angiography guidance [11], especially in patients with simple lesions. The lack of robust evidence supporting the value of IVUS imaging as well as the fact that IVUS increases considerably procedure time and cost have restricted the clinical applications of this modality. However, recent meta-analyses comparing outcomes between patients undergoing IVUS-guided PCI versus patients undergoing angiography-guided PCI have showed significantly low rate of MACE, in the IVUS-guided group [7]. The results reported in the present analysis are agreement with those reported in previous studies, showing that IVUS may play a fundamental role in the treatment of patients with coronary artery disease as it significantly reduces clinical adverse events.

Potential differences in the baseline characteristics of the patients recruited in each study are likely to introduce bias and affect the reported results. To address this limitation we performed repeated analysis in propensity-matched and randomized populations. Of note, the repeated results confirmed that IVUS guidance increased safety and efficacy during the PCI. In the randomized AVIO trial [11], the occurrence of cumulative MACE in the IVUS guided group was apparently lower than the angiography guided group (16.9 % vs. 23.2 %) at 2 years follow-up. Although the study failed to show statistical significant differences in this composite endpoint, this should be predominantly attributed to the limited sample size (*n* = 284).

In the present meta-analysis we found an increased beneficial effect of IVUS guidance in complex lesions and in patients admitted with ACS with respect to death. Similarly, in the substudy of ADAPT-DES (Assessment of Dual Antiplatelet Therapy With Drug-Eluting Stents), the benefits of IVUS in reducing MACE were more evident in patients with ACS and complex lesions [4]. Indeed, IVUS is recommended for sizing bifurcation stents and evaluating lesion severity in the consensus documents from European bifurcation club [34]. Apart from this, IVUS also has an accurate correlation between IVUS derived minimal area and fractional flow reserve (FFR) to facilitate detection of hemodynamically significant left main lesions [35]. A recent large registry that recruited patients who had unprotected left main PCI showed that IVUS guidance was associated with significant reductions of 1-year cardiac death (1.7 % vs.5.2 %, *p* = 0.023), TVR (3.4 % vs. 10.0 %, *p* = 0.002) and MACE (16.2 % vs. 24.4 %, *p* = 0.014) [28]. Consistently, a recent study from Europe also showed a low rate of MACE (11.7 % vs. 16 %, *p* = 0.04) in patients with left main coronary disease having IVUS guided PCI [26]. In the present sub-group analysis for patients with complex lesions or ACS, we included studies with IVUS guided PCI for left main stem disease, bifurcation, CTO, small vessel, long lesion, and ACS. Although some studies have also reported opposing results, there was a significant favorable effect of IVUS guidance on clinical outcomes in this subset of patient populations [24, 25, 28].

Recently, Fröhlich GM et al. reported the long-term survival of a large cohort study (Angiography versus IVUS or intracoronary pressure wire-derived measurements of FFR to guide elective or urgent PCI) in patients undergoing PCI at eight London centers between 2004 and 2011 (*n* = 41,688) [5]. Surprisingly, patients who underwent IVUS had a higher adjusted mortality compared with angiography guided PCI (hazard ratio: 1.39; 95 % CI: 1.09-1.78; *P* = 0.009), although this difference was no longer statistically significant in a matched pair of 803 patients. Obviously, the adjusted analysis is likely to introduce an error; in addition the absence of important procedural and lesion characteristics which may have a critical impact on clinical outcomes did not allow drawing safe conclusions. Moreover, the absence of pre-specified criteria for IVUS imaging is also likely to have affected the reported results. These limitations could potentially explain the discrepancy noted between this study and our analysis which included a large sample size, the sub-analysis of matched and randomized studies, and the stratified analysis on complex lesions or ACS, which showed that IVUS guided PCI reduces death, ST, and MACE at a mean weighted follow-up of 20.8 months.

The results of the present meta-analysis and the inconsistent findings of previous studies underscore the need to design a large randomized control trial that would have enough power to investigate the efficacy of IVUS guided PCI in the 2^nd^ generation DES era. Certainly, a cost-effectiveness analysis of IVUS use during PCI should be incorporated into the additional benefit on clinical outcomes.

### Limitations

Our study has several limitations. It is a meta-analysis and shares the limitations from the original studies. The inability to adjust the baseline characteristics between the 2 studied groups may introduce remarkable bias. However, the findings consistently showed that IVUS guidance was associated with improved outcomes in the all included studies and the repeated analysis that included matched and randomized studies. The current study was not able to differentiate the impact of IVUS guidance in patients treated with either first or newer generation DES.

## Conclusions

IVUS guided PCI was associated with better clinical outcomes than angiography guided DES implantation. The benefit appeared more significant in the subgroup of patients with complex lesions or ACS with respect to death. Large scale randomized control trials are needed to identify which types of lesion morphology and subgroups of patients will be associated with better clinical outcomes after the IVUS guided DES implantation.

## Abbreviations

IVUS: Intravascular ultrasound
DES: Drug-eluting stents
OR: Odds ratios
CI: Confidence intervals
ST: Stent thrombosis
ADAPT-DES: Assessment of Dual Antiplatelet Therapy With Drug-Eluting Stents
MI: Myocardial infarction
MACE: Major adverse cardiac events
PCI: Percutaneous coronary intervention
ACS: Acute coronary syndromes
PRISMA: Preferred Reporting Items for Systematic Reviews and Meta-analyses
BMS: Bare metal stents
NOS: Newcastle-Ottawa-Scale
TVR: Target vessel revascularization
TLR: Target lesion revascularization
CTO: Chronic total occlusion
MLD: Minimum luminal diameter
FFR: Fractional flow reserve
